# The Role of Artificial Intelligence Chatbots in Chronic Disease Care: A Systematic Review of Current Evidence and Future Directions

**DOI:** 10.7759/cureus.111334

**Published:** 2026-06-22

**Authors:** Mohamed Abdalla Mohamed ElshikhIdris, Asjed Salaheldin Hassan Ali, Sara Y Mahmoud, Yousra Mahdi Salih Aziz, Inas Ali, Salma Farah, Wafa Farid Mohamed Altayeb

**Affiliations:** 1 General Practice, Al Yarmouk College, Khartoum, SDN; 2 Internal Medicine, University of Khartoum, Khartoum, SDN; 3 Geriatrics, Sandwell and West Birmingham Hospitals/Midland Metropolitan University Hospital (MMUH), Birmingham, GBR; 4 General Practice, Omdurman Islamic University, Khartoum, SDN; 5 Cardiology, South Tipperary University Hospital, Gortmaloge, IRL; 6 Palliative Care Medicine, University Hospital Galway, Galway, IRL; 7 Acute Medicine, Royal Stoke University Hospital, Newcastle Under Lyme, GBR

**Keywords:** artificial intelligence, chatbots, chronic disease, conversational agents, digital health, self-management, systematic review

## Abstract

Chronic diseases impose a substantial burden on healthcare systems, requiring innovative approaches to support long-term self-management. AI-driven chatbots have emerged as a scalable digital health intervention, but the evidence base remains fragmented. This systematic review synthesizes current evidence on the role of AI chatbots in chronic disease care and evaluates their impact on clinical and behavioural outcomes.

A comprehensive literature search was conducted in PubMed, Embase, Scopus, and Web of Science for studies published between 2021 and 2025. The review followed PRISMA (Preferred Reporting Items for Systematic Reviews and Meta-Analyses) 2020 guidelines, and the narrative synthesis was reported in accordance with the Synthesis Without Meta-analysis (SWiM) guidelines; the certainty of the evidence was rated using the GRADE (Grading of Recommendations Assessment, Development, and Evaluation) approach. Risk of bias was assessed using the Cochrane RoB 2 tool for RCTs and the Joanna Briggs Institute (JBI) Critical Appraisal Checklist for non-randomized studies (inter-reviewer agreement Cohen κ = 0.84-0.90). Eleven studies met the inclusion criteria, covering conditions such as diabetes, hypertension, asthma, cancer, mental health disorders, and smoking cessation. Chatbot technologies ranged from rule-based systems to advanced natural language processing (NLP) and large language models (LLMs). AI chatbots improved smoking cessation rates (26% vs. 18.8%; absolute difference 7.2 percentage points), reduced depression and anxiety in some studies, and demonstrated high usability and acceptability. No serious adverse events were reported. However, methodological limitations included small sample sizes, short follow-up periods, high attrition, and the absence of control groups, and the overall certainty of the evidence was low to very low across outcome domains. AI chatbots are feasible and acceptable for chronic disease care, particularly for smoking cessation and mental health support; however, current evidence is insufficient to confirm effects on hard clinical endpoints. Future research requires large-scale RCTs with objective clinical endpoints and longer follow-up.

## Introduction and background

Chronic diseases such as diabetes mellitus, cardiovascular disease, chronic respiratory conditions, and chronic kidney disease represent a major and growing global health burden [1]. These conditions require long-term, continuous management, often placing substantial demands on healthcare systems and patients alike. Effective management depends not only on pharmacological treatment but also on sustained patient engagement, adherence to lifestyle modifications, self-monitoring, and timely clinical support [2]. However, traditional healthcare delivery models frequently struggle to provide the level of continuous, personalized, and scalable care needed to optimize outcomes in chronic disease populations, particularly in resource-limited settings [3].

In recent years, artificial intelligence (AI)-driven chatbots have emerged as a promising digital health intervention designed to bridge this gap [4]. These systems leverage natural language processing (NLP), machine learning, and rule-based algorithms to simulate human-like conversations and deliver real-time, interactive support to patients [5]. In chronic disease care, chatbots have been deployed for a variety of functions, including medication reminders, symptom monitoring, lifestyle coaching, psychological support, and patient education. Their ability to provide 24/7 accessibility, scalability, and low-cost interaction positions them as potentially transformative tools in enhancing self-management and extending the reach of healthcare services beyond traditional clinical settings [6].

AI chatbots span a spectrum of architectures: rule-based (decision-tree) systems follow predefined scripts and fixed inputs; NLP- and machine-learning-based systems interpret free text using statistical language models, and large language model (LLM)-based systems (e.g., the GPT and Gemini families) generate fluent, context-aware responses without task-specific scripting [7]. In chronic disease care, these tools deliver glucose-monitoring reminders in diabetes, blood-pressure self-measurement and adherence prompts in hypertension, cognitive behavioural therapy (CBT)-based support in mental health, and behavioural coaching for smoking cessation. Their uptake has accelerated with the post-COVID-19 expansion of telemedicine and, since 2022, with the public release of generative LLMs [7].

This rapid adoption also raises concerns that warrant systematic evaluation: chatbots process sensitive health data (privacy and security risks), generative systems can produce inaccurate or fabricated advice (“hallucinations”), and reliance on smartphones may exclude patients with limited digital literacy, potentially widening inequities.

Despite the growing enthusiasm and rapid adoption of AI chatbots in healthcare, the current evidence base remains fragmented, with considerable heterogeneity in study designs, populations, intervention types, and reported outcomes. Questions persist regarding their true clinical effectiveness, long-term sustainability, user engagement, and safety across different chronic disease contexts.

Prior reviews are largely dated or narrow, evaluating mainly rule-based or single-condition (often mental-health) agents [6,8], with a recent review limited to behaviour-change applications [9] and the only chronic-illness synthesis predating the post-2023 generative-AI wave [5]. No current review spans the full architectural spectrum and breadth of chronic conditions while appraising methodological quality and certainty of evidence - the gap this review addresses.

Therefore, this systematic review aims to critically synthesize the existing literature on AI chatbot interventions in chronic disease care, evaluate their impact on clinical and behavioural outcomes, and identify key limitations and future research directions to guide the development of more effective, equitable, and evidence-based digital health solutions.

## Review

Methodology

Study Design and Aim

This systematic review was conducted in accordance with the Preferred Reporting Items for Systematic Reviews and Meta-Analyses (PRISMA) 2020 guidelines [10] to ensure transparency, reproducibility, and methodological rigour. The review aimed to systematically identify, appraise, and synthesize evidence on the role of AI chatbots in chronic disease care, focusing on their clinical, behavioural, and patient-reported outcomes. A review protocol was not prospectively registered, and this is acknowledged as a limitation.

Eligibility Criteria

Study eligibility was defined using the PICOS framework [11]. The Population (P) included adult or pediatric patients diagnosed with any chronic disease, such as diabetes mellitus, cardiovascular disease, chronic respiratory diseases, chronic kidney disease, or other long-term non-communicable conditions. The Intervention (I) comprised AI-based chatbot systems, conversational agents, or virtual assistants designed to support disease management, self-care, or patient engagement. The Comparison (C) included usual care, standard digital interventions, or no intervention, where applicable. The Outcomes (O) included clinical outcomes (e.g., HbA1c, blood pressure, symptom control), behavioural outcomes (e.g., medication adherence, lifestyle modification), and patient-reported outcomes (e.g., satisfaction, engagement, quality of life). The Study design (S) included randomized controlled trials (RCTs), quasi-experimental studies, cohort studies, and other original empirical studies, while reviews, editorials, conference abstracts without full data, and non-human studies were excluded.

To make the intervention criterion explicit, eligible conversational agents could be of any architecture - rule-based/decision-tree systems, NLP- or machine-learning-based agents, or LLM-based generative chatbots - delivered via text or voice. Hybrid human-in-the-loop systems were eligible, provided the conversational agent was the primary mode of delivery. Stand-alone symptom-checkers or triage tools without a self-management or patient-support function, and interventions targeting acute (non-chronic) conditions, were excluded. Accordingly, one report addressing COVID-19 testing behaviour, an acute rather than chronic indication, was excluded at full-text review even though it employed a chatbot.

Information Sources

A comprehensive literature search was conducted in four major electronic databases: PubMed, Embase, Scopus, and Web of Science. These databases were selected due to their extensive coverage of biomedical, clinical, and interdisciplinary digital health research, ensuring broad retrieval of relevant studies on AI-driven healthcare interventions.

Search Strategy

A systematic search strategy was developed using a combination of controlled vocabulary (e.g., MeSH terms) and free-text keywords related to “artificial intelligence”, “chatbots”, “conversational agents”, and “chronic disease management”. Search terms explicitly included generative AI and LLM vocabulary (e.g., “large language model”, “GPT”, “ChatGPT”, “generative AI”) to ensure that recent LLM-based systems were captured. Boolean operators (AND/OR) were applied to combine search concepts appropriately. The search strategy was adapted for each database to account for differences in indexing and syntax. To ensure the inclusion of the most current and relevant evidence, the search was limited to studies published between 2021 and 2025. This timeframe was selected because AI chatbot technologies have evolved rapidly in recent years, particularly with advances in large language models and NLP, making earlier studies less representative of current clinical applications and technological capabilities. The full database-specific search strings, including all controlled-vocabulary and free-text terms, Boolean combinations, applied filters, and the exact dates of the search, are provided in Appendix 1 to support reproducibility.

Study Selection

All retrieved records were imported into EndNote X21 for reference management and duplicate removal. After deduplication, studies were screened in two stages: first by title and abstract, and then by full-text review against the predefined eligibility criteria. Two independent reviewers performed the screening, and any disagreements were resolved through discussion or consultation with a third reviewer to ensure consistency and reduce selection bias. Inter-reviewer agreement was quantified using Cohen κ and was substantial to high at both stages (title/abstract screening, κ = 0.84; full-text selection, κ = 0.89). Disagreements that could not be resolved by discussion (n = 6 at the full-text stage) were adjudicated by a third reviewer.

Data Extraction

Data extraction was conducted using a standardized extraction form designed for this review. Extracted variables included study characteristics (author, year, country, design), population details, type of chatbot intervention and architecture, chronic disease focus, duration of intervention, comparator details, and reported outcomes (including effect estimates, confidence intervals, and p-values where available). Additional information on key findings and limitations was also collected to support qualitative synthesis. Extraction was performed independently by two reviewers and cross-checked, with agreement on key variables of κ = 0.90.

Risk of Bias and Certainty Assessment

The methodological quality and risk of bias of the included studies were assessed using appropriate, validated tools based on study design. RCTs were evaluated using the Cochrane Risk of Bias 2 (RoB 2) tool [12], while observational and non-randomized studies were assessed using the Joanna Briggs Institute (JBI) Critical Appraisal Checklists [13]. Each study was independently appraised by two reviewers, and discrepancies were resolved through consensus to ensure reliability and minimize subjective bias in quality assessment.

Prespecified decision rules were applied. For RCTs, the overall RoB 2 rating followed the tool’s algorithm (low risk only if all domains were low; high risk if any domain was high; otherwise, some concerns). For non-randomized studies, JBI items were rated yes/no/unclear and summarized as high (≥70% of items met), moderate (50-69%), or poor (<50%) quality. Certainty of evidence for each outcome domain was rated using GRADE (Grading of Recommendations Assessment, Development, and Evaluation) [14], considering risk of bias, inconsistency, indirectness, imprecision, and publication bias.

Data Synthesis

A narrative synthesis was conducted and reported following the Synthesis Without Meta-analysis (SWiM) guideline [15] and the framework of Popay et al. [16]. Studies were grouped by outcome domain (behavioural; mental-health/psychological; physiological/disease-specific; and usability/acceptability) and, within domains, by chatbot architecture and condition; for each domain, the direction and - where reported - the magnitude of effect (with confidence intervals and p-values) were summarized and assessed for consistency.

A meta-analysis was not performed because of substantial clinical and methodological heterogeneity - differing study designs, outcome definitions, measurement tools (e.g., different scales for adherence or quality of life), and intervention durations - and because many studies reported insufficient statistical data (e.g., missing standard deviations or effect sizes) for meaningful pooling.

Pooling outcome subsets was also not feasible: only single trials addressed smoking cessation and blood pressure, and the mental-health studies used different instruments. Because each outcome included fewer than 10 comparable studies, formal publication-bias tests (e.g., funnel plots, Egger’s test) were inappropriate, so publication bias is addressed qualitatively.

Results

Study Selection

**Figure 1:**
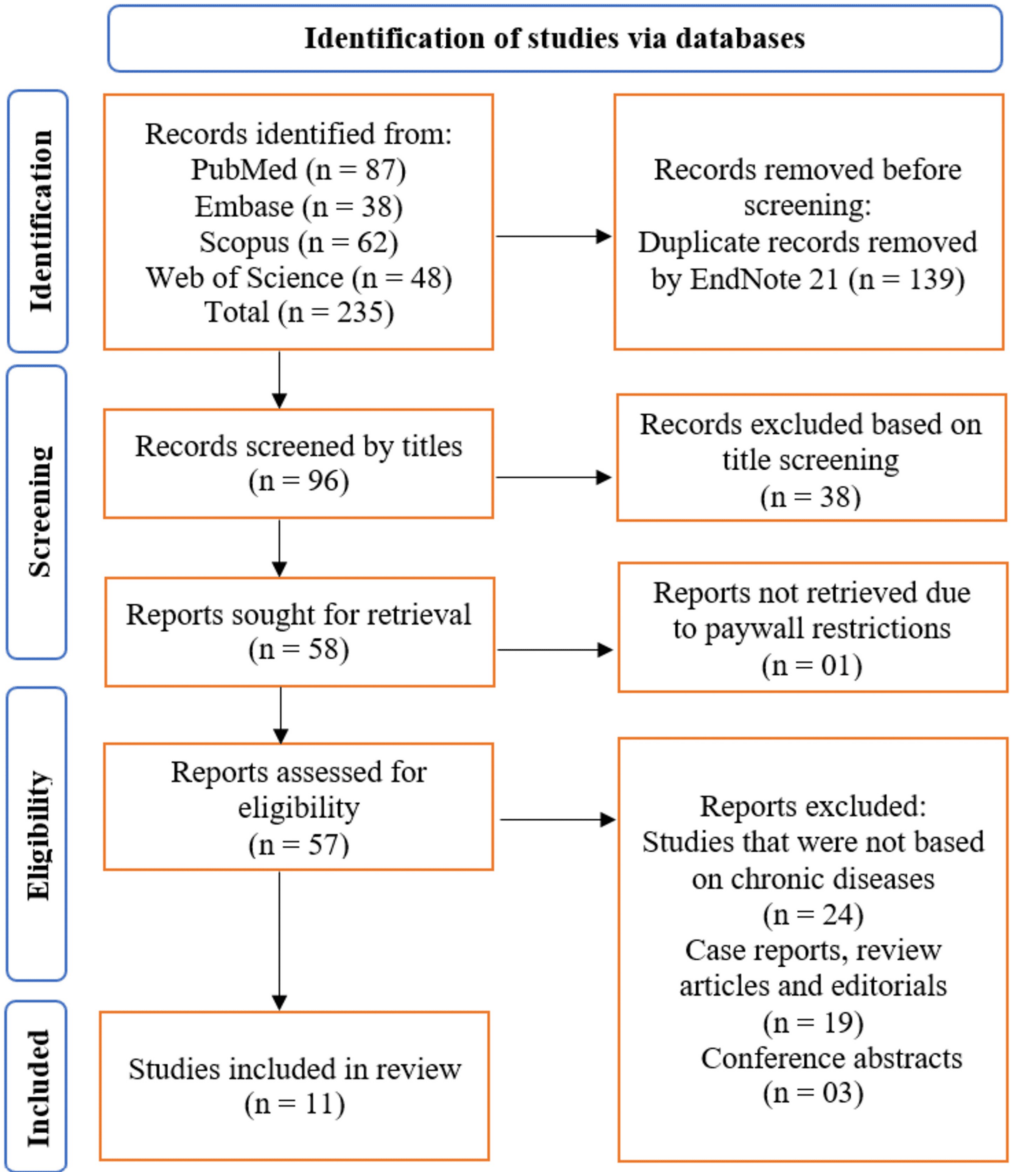
Studies Selection Process (PRISMA Flowchart) PRISMA: Preferred Reporting Items for Systematic Reviews and Meta-Analyses

A total of 235 records were identified through searches across four electronic databases: PubMed (n = 87), Embase (n = 38), Scopus (n = 62), and Web of Science (n = 48). After removing duplicate records using EndNote 21 (Clarivate, Philadelphia, Pennsylvania) (n = 139), 96 unique records remained for title screening. Following title screening, 38 records were excluded, yielding 58 reports sought for retrieval. One report could not be retrieved due to paywall restrictions, leaving 57 reports assessed for full-text eligibility. Of these, 46 reports were excluded for the following reasons: studies that were not based on chronic diseases (n = 24, including one report addressing COVID-19 testing behaviour, an acute rather than chronic indication, which was excluded as outside the chronic disease scope of this review), case reports, review articles, and editorials (n = 19), and conference abstracts (n = 3). Consequently, 11 studies [17-27] met the inclusion criteria and were included in this systematic review (Figure 1).

Study Characteristics

Across the 11 included studies, study designs and populations were highly heterogeneous, reflecting an emerging field. The studies were published between 2021 and 2025, with the majority appearing in JMIR-series journals. Geographically, the studies were conducted across nine countries: South Korea [17], Canada [18,21], New Zealand [19], Spain [20,26], Italy [22], South Africa [23], Norway [24], Switzerland [25], and France [27]. Study designs varied considerably, including RCTs [18,20,21,26], pilot and pragmatic trials [20,25], mixed-methods studies [19,23,27], development and usability studies [17], observational log analyses [24], feasibility studies [25], and proof-of-concept pre-post designs [22]. Sample sizes ranged from 13 participants [22] to over 8,000 individuals [23]. Chronic diseases addressed included multiple chronic conditions [17], arthritis and diabetes [18], health anxiety [19], smoking cessation [20], cancer [21], type 1 and type 2 diabetes mellitus [22,23], gestational diabetes mellitus [24], pediatric asthma [25], hypertension with or without chronic kidney disease [26], and sickle cell disease [27]. Full characteristics are summarized in Table 1.

**Table 1:** Characteristics of Included Studies AI = artificial intelligence; AYA = adolescents and young adults; BP = blood pressure; CBT = cognitive behavioural therapy; CKD = chronic kidney disease; COVID-19 = coronavirus disease 2019; DM = diabetes mellitus; F = female; FU = follow-up; GDM = gestational diabetes mellitus; GPT-2 = generative pre-trained transformer 2; HTN = hypertension; Jul = July; min = minutes; NLP = natural language processing; OA = osteoarthritis; OSG = online support group; pre–post = before-and-after study design; pts = patients; RA = rheumatoid arthritis; RCT = randomized controlled trial; T1DM = type 1 diabetes mellitus; T2DM = type 2 diabetes mellitus; VC = virtual coach; vs. = versus; y = years; ≥ = greater than or equal to; ± = with or without.

Author (Year)	Country	Study Design	Sample Size	Chronic Disease Focus	Participant Characteristics	Intervention Duration	AI Chatbot Type	Comparator
Zhang and Song, [17] (2024)	South Korea	Development + cross-sectional usability	64 users	24 chronic diseases	Adults; Chinese; mixed education; non-patients	1 session	GPT-2 chatbot (Chat Ella)	None
MacNeill et al., [18] (2024)	Canada	RCT (mixed-methods)	68	Arthritis, Diabetes	Adults 19–65y; mean 42.9y; 69% F	4 weeks	Wysa (CBT-based)	Waitlist / no intervention
Goonesekera and Donkin, [19] (2022)	New Zealand	Mixed-method pilot	69 enrolled; 29 analyzed	Health anxiety	Adults ≥18; mostly female; social-media recruited	14 days	CBT decision-tree chatbot (“Otis”)	None (pre–post)
Olano-Espinosa et al., [20] (2022)	Spain	Pragmatic multicenter RCT	513	Smoking cessation	Adult smokers (>18), willing to quit	6 months	Telegram AI chatbot (Bayesian/NLP)	Usual primary care counselling
Leung et al., [21] (2022)	Canada	Pilot RCT	48	Cancer	Adult cancer patients; mostly female	6–8 sessions	NLP recommender (CancerChatCanada AICF)	Therapist-led OSG (no AI)
Bassi et al., [22] (2022)	Italy	Pilot pre–post	13	T1DM & T2DM (psychosocial)	Adults 18–51y; 77% F	12 sessions + 2-mo FU	NLP virtual coach (Motibot, Telegram)	None
Mash et al., [23] (2022)	South Africa	Mixed-methods implementation	8158 (4577 analyzed)	Type 2 DM	Adults, mean 51y, 56% F, public sector	6 months	WhatsApp AI chatbot (GREAT4Diabetes)	None
Sagstad et al., [24] (2022)	Norway	Observational (log analysis)	610 dialogues	GDM	Pregnant women with GDM	20 weeks	Dina (rule-based info chatbot)	None
Kowatsch et al., [25] (2021)	Switzerland	Single-arm feasibility	49	Asthma (children)	10–15 yrs; asthma patients + family	~3–4 weeks	MAX AI chatbot (hybrid, human-in-loop)	None
Echeazarra et al., [26] (2021)	Spain	RCT (2-arm)	112 (88 analyzed)	HTN ± CKD	Adults ≥18; BP self-monitoring	7 days	TensioBot (Telegram bot)	Paper instructions + usual nurse care
Issom et al., [27] (2021)	France	Mixed-methods	33	Sickle Cell Disease	AYA ≥16y, ~37y mean, 66.7% F	~45-min session + survey	Facebook Messenger chatbot (TREVOR, rule-based)	None

Chatbot Technologies and Intervention Design

The AI chatbot technologies employed ranged from relatively simple rule-based systems to more advanced NLP and machine-learning models. Rule-based chatbots were used in several studies [19,26,27], often built on platforms such as Chatfuel [19], Telegram bots [26], or Facebook Messenger [27]. More advanced NLP-driven systems included fine-tuned transformer models such as GPT-2 [17], Bayesian and NLP hybrid systems [20], Rasa-based machine-learning architectures [22], and word2vec-based recommender systems [21]. One study used a hybrid model incorporating human-in-the-loop support [25]. The mode of interaction was predominantly text-based chat, delivered via popular messaging platforms including Facebook Messenger [19,27], Telegram [20,22], WhatsApp [23], and custom mobile or web applications [17,18,21,24-26]. Several chatbots incorporated additional features such as buttons, scripted replies, reminders, alerts, multimedia content, and email follow-ups to enhance engagement [19,21,23,26]. Technological architectures, interaction modes, target functions, and key outcomes are summarized in Table 2. Notably, the included interventions spanned the full architectural spectrum defined in the Introduction (rule-based, NLP/machine learning, and early generative (GPT-2) systems), allowing comparison of outcomes across chatbot types.

**Table 2:** Chatbot Intervention Features and Clinical Outcomes ↑ = increased or improved; ↓ = decreased or reduced; ~ = approximately; > = greater than; ± = plus or minus; % = percent; ABPM = ambulatory blood pressure monitoring; Acc = accuracy; AE = adverse event; AI = artificial intelligence; AUC = area under the curve; BP = blood pressure; CBT = cognitive behavioural therapy; COVID-19 = coronavirus disease 2019; CUQ = Chatbot Usability Questionnaire; e.g. = exempli gratia (for example); et al. = et alii (and others); eval = evaluation; F1 = F1 score (harmonic mean of precision and recall); GAD-7 = Generalized Anxiety Disorder 7-item scale; GDM = gestational diabetes mellitus; GPT-2 = generative pre-trained transformer 2; HTN = hypertension; IUS-12 = Intolerance of Uncertainty Scale 12-item version; min = minutes; ML = machine learning; mo = months; n = sample size; NLP = natural language processing; NS = not statistically significant; ONS4 = Overall Neuroticism Scale 4-item version; OSG = online support group; p = p-value (probability value); PAID-5 = Problem Areas in Diabetes 5-item scale; PHQ-9 = Patient Health Questionnaire 9-item scale; pos = positive; Prec = precision; PSS-10 = Perceived Stress Scale 10-item version; pts = patients; QoL = quality of life; Qs = questions; RCT = randomized controlled trial; Rec = recall; SCD = sickle cell disease; SHAI-18 = Short Health Anxiety Inventory 18-item version; sig. = significant; SMS = short message service (text messaging); SUS = System Usability Scale; TTMC = talk-to-machine-in-Chinese (or as specified in original study); UX = user experience; USE = Usefulness, Satisfaction, and Ease of use questionnaire; vs. = versus; VOC = vaso-occlusive crisis; WHO-5 = World Health Organization Five Well-Being Index; word2vec = word to vector (natural language processing technique).

Author (Year)	Technology Type	Mode of Interaction	Target Function	Duration of Use	Key Outcomes Measured	Main Findings	Adverse Events / Limitations
Zhang and Song, [17] (2024)	GPT-2 (fine-tuned transformer); Flask; React	Text chat (symptoms→diagnosis)	Chronic disease prediction (24 diseases)	1-week usability test (n=64)	AUC, Acc, Prec, Rec, F1, CUQ	High performance (AUC~1; all >0.97); CUQ 68.31; good usability	Non-clinical dataset; small sample; limited real-world validation
MacNeill et al., [18] (2024)	AI CBT-based chatbot (free + scripted)	Text chat	Mental health support in arthritis & diabetes	4 weeks	PHQ-9, GAD-7, PSS-10	↓ depression & anxiety; no stress change	Small sample (n=68), short duration, tech issues, limited personalization
Goonesekera and Donkin, [19] (2022)	Rule-based CBT chatbot (Chatfuel)	Messenger app; guided replies + reminders	14-day CBT for health anxiety	14 days (+ up to 21 days access)	SHAI-18, GAD-7, IUS-12, ONS4, WHO-5, adherence	No change in health anxiety; ↓ GAD-7; ↑ well-being & QoL; high acceptability	Technical issues, limited personalization, time burden, attrition
Olano-Espinosa et al., [20] (2022)	AI expert system (NLP + Bayesian + gamification)	Text chatbot via Telegram	Smoking cessation support	6 months	6-mo abstinence, QoL, contacts, interaction time	Higher abstinence vs. control (26% vs. 18.8%); intensive use improved success	No AEs reported; high dropout; no user feedback
Leung et al., [21] (2022)	NLP (word2vec), recommender system	Text-based OSG + post-session email	Detect psychosocial concerns; recommend resources	6–8 sessions	Precision, recall, F1, engagement	F1 improved 0.571→0.880; high recall (0.981); 52% clicked resources; 76% useful	English-only; misclassification; early false pos/neg; small sample
Bassi et al., [22] (2022)	ML-based NLP (Rasa, TTMC)	Text + buttons via Telegram	Psychosocial support	12 sessions (10–20 min) + 2-mo FU	PHQ-9, GAD-7, PSS-10, PAID-5, WHO-5, UX	No sig. change; ↓ trend; high UX; 77% reported symptom relief	Small sample (n=13), no control, short-term, self-report bias
Mash et al., [23] (2022)	WhatsApp automated (audio/text/image flow)	Interactive WhatsApp + feedback	Diabetes self-management education	6 months	Acceptability, reach, behaviour, confidence, cost	High reach; >90% usefulness; 71% behaviour change; 87.6% confidence gain; low completion (12.6%)	Dropout, tech errors, access barriers, scalability issues
Sagstad et al., [24] (2022)	Web-based, user-centred	Text + buttons, no login	GDM education & self-care	20-week log study	Dialogues, Qs, usage time, fallback rate	610 dialogues, 88% answered; most Qs glucose/diet; better navigation over time	No user-ID tracking; language barriers; observational design
Kowatsch et al., [25] (2021)	Hybrid AI (app/SMS/web; human-in-loop)	Chat + SMS + email	Pediatric asthma self-care	30 days (14 sessions)	Reach, alliance, knowledge, inhalation, completion	49.5% reach; ↑ knowledge; ↑ inhalation skill; high acceptance	Small, single-arm, dropout, device barriers
Echeazarra et al., [26] (2021)	Mobile chatbot (rule-based)	Text commands, buttons, alerts, video	BP monitoring, education, adherence, reminders	7-day monitoring cycles	BP knowledge, accuracy vs. ABPM, adherence, satisfaction	↑ knowledge (p≈0.04); BP agreement (NS); high satisfaction; improved adherence	No AEs; small sample, partial follow-up, short window
Issom et al., [27] (2021)	Rule-based chatbot	Text + media	SCD self-care, VOC prevention, education	45 min + survey	SUS, USE, usability, feedback	SUS 83 (high); USE 25/36; good usability, empathy, acceptance	Bugs (specific devices), rigid responses, readability, short-term eval

Synthesis of Clinical and Behavioural Outcomes

Behavioural outcomes: smoking cessation and self-management: Behavioural outcomes were the domain with the clearest signal of benefit. One large pragmatic multicenter RCT [20] evaluated a Telegram-based AI chatbot for smoking cessation in 513 adult smokers; at six months, the chatbot group achieved a significantly higher abstinence rate (26%) than usual primary care counselling (18.8%), an absolute difference of 7.2 percentage points, with more intensive use associated with greater success. In diabetes self-management, a WhatsApp chatbot (GREAT4Diabetes) delivered to over 8,000 public-sector patients [23] reported high acceptability, with 71% of users showing positive behaviour change and 87.6% reporting increased confidence in self-management, although intervention completion was low (12.6%). Across these behavioural studies, chatbots consistently improved self-reported behaviour and engagement, but only the smoking-cessation trial provided a controlled, objective endpoint.

Mental-health and psychological outcomes: Mental-health findings were mixed and instrument-dependent. A four-week RCT using a CBT-based chatbot (Wysa) in 68 adults with arthritis or diabetes [18] significantly reduced depression and anxiety scores versus a waitlist control, with no significant change in stress. In contrast, a 14-day CBT chatbot (Otis) [19] did not reduce health anxiety (SHAI-18) but did reduce generalized anxiety (GAD-7) and improved well-being and quality of life. A proof-of-concept study of an NLP-based virtual coach (Motibot) for adults with diabetes [22] found no significant change in depression, anxiety, or stress over 12 sessions, although 77% of participants self-reported symptom relief and engagement was high. The divergent results likely reflect differences in intervention duration, outcome instruments, and baseline symptom severity rather than a uniform class effect.

Physiological and disease-specific outcomes: Effects on physiological and disease-specific endpoints were weaker and largely confined to knowledge and self-management rather than hard clinical measures. A rule-based chatbot for blood-pressure monitoring (TensioBot) in 112 adults with hypertension (± CKD) [26] significantly improved blood-pressure knowledge (p≈0.04) and adherence over a 7-day period but did not change blood-pressure agreement with ambulatory monitoring. A gestational diabetes chatbot [24] answered 88% of 610 dialogues, with most queries concerning glucose monitoring and diet, though its observational design precluded causal inference. A pediatric-asthma feasibility study [25] reported improved inhalation skills and asthma knowledge, limited by a single-arm design and high attrition.

Diagnostic performance and cancer support: An NLP-based recommender system (CancerChatCanada AICF) [21] detected psychosocial challenges in cancer patients, improving its F1 score from 0.571 to 0.880 (recall 0.981); over half of participants clicked recommended resources and 76% found them useful, although early false positives/negatives and a small sample limited generalizability. A GPT-2-based chatbot for auxiliary diagnosis of 24 chronic diseases [17] achieved very high performance metrics (AUC ~1.00; accuracy, precision, and recall all >0.97) and good usability (CUQ 68.31), but relied on a non-clinical dataset and a small sample, limiting real-world validity.

Usability and Acceptability

Usability and acceptability were consistently high across architectures and conditions. Reported metrics included a System Usability Scale score of 83 for a rule-based sickle-cell chatbot [27], a Chatbot Usability Questionnaire score of 68.31 for the GPT-2 diagnostic system [17], and satisfaction ratings exceeding 90% for the blood-pressure chatbot [26]. Several studies reported high engagement and perceived empathy even where clinical effects were small [19,22], suggesting that acceptability is not, by itself, a sufficient indicator of clinical benefit.

Safety and Adverse Events

Across all 11 studies, no serious adverse events directly attributable to AI chatbot interventions were reported, and two trials [20,26] explicitly stated that none occurred. However, several studies documented technical issues, including device-specific bugs [27], rigid or unhelpful responses [27], device barriers [25], language barriers [24], and scalability challenges [23]. Common methodological limitations included small sample sizes [18,19,21,22,26], short intervention or follow-up durations [22,26,27], high attrition or dropout [20,23], lack of control groups [17,19,22-25,27], and limited personalization or real-world validation [18,19,21]. Harms were generally not assessed using standardized frameworks, so the apparent absence of adverse events should be interpreted cautiously.

Summary of Reported Effect Estimates

To make the available quantitative evidence explicit, reported effect estimates are collated in Table 3. Most studies reported direction of effect and, for behavioural and physiological outcomes, point estimates or p-values; however, few reported confidence intervals, and outcomes were measured with heterogeneous instruments, precluding pooling (see Methods).

**Table 3:** Summary of Reported Effect Estimates Across Included Studies PHQ-9 = Patient Health Questionnaire 9-item scale; GAD-7 = Generalized Anxiety Disorder 7-item scale; WHO-5 = World Health Organization Five Well-Being Index; CI = confidence interval; PSS-10 = Perceived Stress Scale 10-item version; AUC = area under the curve; BP = blood pressure; SUS = System Usability Scale; CUQ = Chatbot Usability Questionnaire; ABPM = ambulatory blood pressure monitoring.

Study	Outcome Domain	Outcome Measure	Effect Estimate (95% CI / p, As Reported)	Direction/Significance
Olano-Espinosa [20]	Behavioural (smoking)	6-month continuous abstinence	26.0% vs. 18.8%; absolute difference 7.2 pp	Favours chatbot (significant)
Mash [23]	Behavioural (self-mgmt)	Self-reported behaviour change; confidence	71% behaviour change; 87.6% confidence gain (single-arm)	Favours (uncontrolled)
MacNeill [18]	Mental health	PHQ-9 (depression); GAD-7 (anxiety)	Significant reduction vs. waitlist (CIs not reported)	Favours chatbot
MacNeill [18]	Mental health	PSS-10 (stress)	No significant change	No difference
Goonesekera & Donkin [19]	Mental health	SHAI-18 (health anxiety)	No significant change	No difference
Goonesekera & Donkin [19]	Mental health	GAD-7; WHO-5	Significant improvement (pre–post)	Favours (uncontrolled)
Bassi [22]	Mental health	PHQ-9 / GAD-7 / PSS-10	No significant change; downward trend; 77% self-reported relief	No significant difference
Echeazarra [26]	Physiological	BP knowledge	Significant improvement (p≈0.04)	Favours chatbot
Echeazarra [26]	Physiological	BP agreement vs. ABPM	No significant difference	No difference
Leung [21]	Diagnostic / usability	Classifier F1; recall	F1 0.571→0.880; recall 0.981	Performance improved
Zhang & Song [17]	Diagnostic / usability	AUC; accuracy; CUQ	AUC ~1.00; metrics >0.97; CUQ 68.31	High (non-clinical data)
Issom [27]	Usability	System Usability Scale	SUS 83	High usability
Sagstad [24]	Process / usability	Query answer rate	88% of queries answered	Descriptive

Risk of Bias and Certainty of Evidence

The risk of bias for the four RCTs was assessed using the Cochrane RoB 2 tool [18,20,21,26]. As shown in Table 4, three studies were judged to have some concerns overall, while one study was rated as high risk. Each demonstrated low risk in the randomization process and outcome-measurement domains but had some concerns related to missing outcome data or deviations from intended interventions, primarily due to small sample sizes, short follow-up, or technical issues. Olano-Espinosa et al. [20] was rated high risk overall due to high dropout rates and lack of user-feedback data. No trial was rated as low risk across all five domains.

**Table 4:** Risk of Bias Assessment Using the Cochrane RoB 2 Tool (Randomized Controlled Trials)

Study	D1: Randomization	D2: Deviations From Intended Interventions	D3: Missing Outcome Data	D4: Measurement of Outcome	D5: Selection of Reported Result	Overall Risk of Bias
MacNeill et al., [18] (2024)	Low	Low	Some concerns	Low	Low	Some concerns
Olano-Espinosa et al., [20] (2022)	Low	Some concerns	High risk	Low	Low	High risk
Leung et al., [21] (2022)	Some concerns	Some concerns	Some concerns	Some concerns	Low	Some concerns
Echeazarra et al., [26] (2021)	Low	Some concerns	Some concerns	Low	Low	Some concerns

The remaining seven studies (pre-post, cross-sectional, observational, feasibility, and mixed-methods designs) were assessed using the JBI Critical Appraisal Checklist [17,19,22-25,27] (Table 5). One study achieved a high-quality rating, four were rated moderate, and one was rated poor. Issom et al. [27] met all 10 JBI criteria, indicating high quality, whereas Bassi et al. [22] was rated poor owing to small sample size, lack of confounding control, and short follow-up. Overall, the majority of non-randomized studies were of moderate quality, primarily limited by the absence of control groups, attrition, small sample sizes, and unmanaged confounding.

**Table 5:** Risk of Bias Assessment Using the JBI Critical Appraisal Checklist (Non-randomized Studies) SC = some concerns; HA = high attrition; Y = yes; N = no

Study	C1	C2	C3	C4	C5	C6	C7	C8	C9	C10	Overall
Zhang and Song, [17]	Y	Y	Y	SC	N	SC	SC	Y	Y	SC	Moderate
Goonesekera and Donkin, [19]	Y	Y	Y	SC	N	SC	Y	Y	Y	SC	Moderate
Bassi et al., [22]	Y	Y	Y	SC	N	SC	SC	SC	Y	SC	Poor
Mash et al., [23]	Y	Y	Y	Y	Y	Y	Y	Y	Y	Y	Moderate
Sagstad et al., [24]	Y	Y	Y	SC	N	SC	Y	Y	Y	SC	Moderate
Kowatsch et al., [25]	Y	Y	Y	Y	N	Y	Y	HA	Y	Y	Moderate
Issom et al., [27]	Y	Y	Y	Y	Y	Y	Y	Y	Y	Y	High

Certainty of evidence, assessed with GRADE, was low to very low across all outcome domains (Table 6), reflecting predominantly small, short, and uncontrolled studies, heterogeneous outcomes, and imprecision. The clearest evidence - still of low certainty - was for short-term behavioural outcomes (smoking cessation) and for usability and acceptability.

**Table 6:** GRADE Certainty of Evidence by Outcome Domain GRADE = Grading of Recommendations Assessment, Development, and Evaluation; RCT = randomized controlled trial.

Outcome Domain	No. of Studies (Design)	Certainty (GRADE)	Main Reasons for Downgrading	Summary Finding
Smoking cessation	1 RCT [20]	Low	Imprecision (single trial); risk of bias (attrition)	Higher 6-mo abstinence vs. usual care (26% vs. 18.8%)
Mental health / psychological	3 (1 RCT, 2 non-RCT) [18,19,22]	Very low	Risk of bias; inconsistency; imprecision; heterogeneous instruments	Mixed; significant in 1 RCT, partial/none in others
Physiological / disease-specific	3 (1 RCT, 2 non-RCT) [23,25,26]	Very low	Risk of bias; indirectness (knowledge/surrogate outcomes); imprecision	Improved knowledge/adherence; no clear effect on hard endpoints
Usability / acceptability	7 (mixed) [17,19,21,23–25,27]	Low	Risk of bias; no comparator in most studies	Consistently high usability and acceptability
Safety (adverse events)	11 (all)	Low	Heterogeneous/under-reported harms	No serious adverse events reported

Discussion

This systematic review synthesized evidence from 11 studies examining the role of AI chatbots in chronic disease care, encompassing diabetes, hypertension, asthma, cancer, mental health disorders, and smoking cessation. Overall, chatbot interventions were generally acceptable and feasible across diverse populations and settings, but their clinical effectiveness varied considerably depending on the target condition, technological design, and study rigour.

Clinical Effectiveness

The clearest evidence of benefit was for behaviour change, particularly smoking cessation and diabetes self-management. Olano-Espinosa et al. [20] reported a 7.2 percentage-point absolute improvement in six-month abstinence using a Telegram-based chatbot, a clinically meaningful difference consistent with prior meta-analyses of digital smoking-cessation interventions; for example, Whittaker et al. [28] found that mobile phone-based interventions increased quit rates by approximately 6-8% relative to control. Similarly, Mash et al. [23] demonstrated high reach and self-reported behaviour change in over 8,000 patients with type 2 diabetes, although low completion (12.6%) highlights the challenge of sustaining engagement, echoing Greenwood et al. [29], who reported that mobile health interventions for diabetes improve glycemic control primarily when users remain actively engaged.

Physiological and disease-specific outcomes improved less consistently. Echeazarra et al. [26] showed that a rule-based chatbot improved blood-pressure knowledge and adherence but not blood-pressure measurements, a pattern of improved knowledge and self-efficacy without corresponding physiological change that is common in early-stage digital health interventions. Kowatsch et al. [25] similarly reported improved inhalation skills and asthma knowledge in children, but a single-arm design and high attrition limit causal inference. More broadly, most studies measured knowledge, attitudes, usability, or short-term behaviour, and only a few reported physiological measures such as blood pressure [26] or abstinence [20]; the limited evidence for effects on hard clinical endpoints is the principal gap in the current literature.

Behavioural and Psychological Outcomes

In mental health, results were mixed. MacNeill et al. [18] found significant reductions in depression and anxiety using a CBT-based chatbot, whereas Goonesekera and Donkin [19] observed improvements in generalized anxiety but not health anxiety, and Bassi et al. [22] reported no significant change despite high engagement and self-reported symptom relief. These discrepancies likely reflect differences in intervention duration, outcome instruments, and baseline severity, consistent with Gaffney et al. [30], who noted that effect sizes for digital mental-health interventions vary widely with the level of human support, personalization, and symptom severity. The absence of serious adverse events across the included studies [20,26] is reassuring and consistent with the broader literature on conversational agents used as adjunctive tools.

Technological Considerations

The review identified a wide spectrum of chatbot architectures, from simple rule-based systems [19,26,27] to NLP models such as GPT-2 [17] and Rasa-based machine-learning systems [22]. Notably, more sophisticated systems did not consistently outperform simpler ones: Issom et al. [27] achieved high usability (SUS 83) with a rule-based sickle-cell chatbot, while Leung et al. [21] required an NLP recommender to detect psychosocial concerns with acceptable accuracy (F1 0.880). This suggests that optimal design depends on the target function-diagnostic or triage applications [17] benefit from language models that process free text, whereas structured educational or behavioural support may be delivered effectively with simpler, more predictable systems. Laranjo et al. [8] similarly concluded that no single architecture is superior across all contexts and that user-centred design and workflow integration matter more than technological sophistication alone. These observations are reinforced by recent appraisals of generative LLMs in medicine, which emphasize that fluency and benchmark accuracy do not guarantee clinical safety or real-world validity [7].

The diagnostic performance of the GPT-2-based chatbot reported by Zhang and Song [17] (AUC approaching 1.0 for 24 chronic diseases) is striking, but the study used a non-clinical dataset and a small usability sample, raising concerns about overfitting and generalizability. Regulatory bodies such as the FDA and European Commission increasingly require prospective validation of AI-based diagnostic tools in representative populations before deployment. Thus, while LLMs hold promise for auxiliary diagnosis, integration into chronic disease care must proceed cautiously, with rigorous external validation and clinician oversight.

Implementation Challenges

The geographic diversity of the included studies, spanning high-income settings such as Canada and Switzerland and middle-income settings such as South Africa, highlights the global applicability of chatbot interventions, but several studies noted implementation barriers. Mash et al. [23] reported technical errors, access barriers, and scalability challenges in South African public-sector patients, while Issom et al. [27] encountered device-specific bugs in France, showing that digital divides persist even in high-income countries. Sagstad et al. [24] identified language barriers and free-text input issues in a gestational diabetes chatbot, underscoring the need for multilingual capabilities and flexible interaction modes.

User engagement remained a persistent challenge: attrition ranged from 12.6% [23] to over 50% [25], mirroring the high dropout commonly observed in mobile health. Personalization and contextual awareness were frequently desired by users [18,19,27], yet most chatbots used generic, scripted responses, and integration with healthcare providers was often lacking - only one study [25] used a human-in-the-loop model, and only three [18,20,26] compared chatbots against an active comparator. Consistent with Pang et al. [31], standalone chatbots may be less effective than those integrated with clinical care teams, as patients value the reassurance and accountability of human providers. Intervention duration varied from short pilots [19] to six-month programmes [20,23], but longer interventions did not reliably yield better outcomes, and no study systematically compared different intervention lengths, leaving the optimal “dose” of chatbot interaction unknown.

Certainty of Evidence and Publication Bias

When appraised with GRADE, the certainty of evidence was low to very low across all outcome domains (Table 6). Downgrading was driven by within-study risk of bias (small, uncontrolled, short studies), inconsistency (notably for mental-health outcomes), indirectness (reliance on knowledge and surrogate rather than clinical endpoints), and imprecision (few events and wide or unreported confidence intervals). These ratings indicate that, although the direction of effect is often favourable, confidence in the size - and in some cases, the existence - of an effect is limited.

Publication bias is a plausible threat in this rapidly evolving field, where positive feasibility studies may be preferentially published. A formal assessment (e.g., funnel-plot asymmetry or Egger’s test) was not appropriate because each outcome domain contained fewer than ten comparable studies and the included studies were clinically and methodologically heterogeneous, conditions under which such tests are uninformative or misleading. We therefore treat publication bias qualitatively: its likely effect would be to overstate benefit, reinforcing the cautious interpretation above. This limitation is also acknowledged in the Limitations section.

Implications for Clinical Practice

For clinicians and health systems, the current evidence supports AI chatbots as adjuncts that can extend education, self-monitoring support, and behavioural coaching, especially for smoking cessation and diabetes self-management, rather than as substitutes for clinical care. Given low certainty of evidence and unresolved concerns about accuracy, privacy, and equity, chatbots are best deployed within, not outside, established care pathways, with clinician oversight, attention to data governance, and safeguards for patients with limited digital access.

Future Research Directions

Future research should prioritize objective clinical endpoints, longer follow-up (e.g., ≥12 months), and linkage with electronic health records to assess real-world impact, since most current studies measured knowledge, attitudes, or short-term behaviour rather than morbidity or mortality. Building on the recommendations previously summarized in the Conclusion, priorities include (i) large-scale pragmatic RCTs with blinded outcome assessment and active comparators; (ii) systematic, standardized reporting of harms, including potential negative effects such as increased anxiety, over-reliance, or displacement of human care; (iii) evaluation of dose-response relationships using factorial or adaptive designs; (iv) integration with sensor data (e.g., Bluetooth-enabled blood-pressure cuffs or inhalers) and with clinical workflows; and (v) prospective external validation of generative, LLM-based systems before clinical deployment.

Limitations

This systematic review has several limitations. First, the review protocol was not prospectively registered (e.g., in PROSPERO), which may increase the risk of reporting bias, although a predefined PICOS framework and eligibility criteria were applied. Second, despite a comprehensive search across four major databases, relevant studies in non-indexed journals, preprint servers, or grey literature may have been missed. Third, substantial heterogeneity in designs, populations, chatbot technologies, and outcomes precluded meta-analysis and pooled effect estimates. Fourth, the quality of included studies was generally moderate to poor - only one study was rated high quality on the JBI checklist [27], and no RCT was low risk across all RoB 2 domains - and the GRADE certainty of evidence was low to very low across all outcome domains. Fifth, publication bias may be present, as studies with null findings are less likely to be published; for the reasons noted in the Methods and Discussion, this could not be formally tested and is acknowledged qualitatively. Sixth, most studies assessed short-term outcomes, with only two [20,23] following participants for six months and none for ≥12 months. Seventh, the review did not include cost-effectiveness analyses. Finally, the rapid pace of AI advancement means some technologies in the included studies (e.g., GPT-2 [17]) have already been superseded by more capable models such as GPT-4 and beyond, potentially limiting the currency of the findings.

## Conclusions

AI chatbots represent a promising adjunct to chronic disease care, demonstrating feasibility, acceptability, and potential effectiveness across conditions, including smoking cessation, diabetes self-management, mental health support, and hypertension education. However, the current evidence base is characterized by methodological heterogeneity, small sample sizes, short follow-up periods, and a predominance of pilot studies without control groups, with overall certainty of evidence ranging from low to very low. No serious adverse events were reported, suggesting that chatbots are generally safe as supportive tools. In short, AI chatbots are not a replacement for human clinicians but offer a scalable, accessible modality to extend chronic disease care beyond traditional settings; realizing this potential will require the rigorous, longer-term, clinically anchored research outlined under Future Research Directions.

## Appendices

Appendix 1

**Table 7:** Search Strategy for Databases

Database	Search String
PubMed	(("Artificial Intelligence"[Mesh] OR "Natural Language Processing"[Mesh] OR "Machine Learning"[Mesh] OR "artificial intelligence"[tiab] OR AI[tiab] OR chatbot*[tiab] OR "conversational agent*"[tiab] OR "virtual assistant*"[tiab] OR "digital assistant*"[tiab] OR "AI chatbot*"[tiab] OR "intelligent virtual agent*"[tiab] OR "large language model*"[tiab] OR LLM*[tiab] OR ChatGPT[tiab] OR Bard[tiab] OR Gemini[tiab])) AND (("Chronic Disease"[Mesh] OR chronic disease*[tiab] OR chronic illness*[tiab] OR long-term condition*[tiab] OR diabetes[tiab] OR hypertension[tiab] OR cardiovascular disease*[tiab] OR heart failure[tiab] OR chronic kidney disease[tiab] OR CKD[tiab] OR COPD[tiab] OR asthma[tiab] OR cancer[tiab] OR arthritis[tiab] OR obesity[tiab])) AND (("Patient Care"[Mesh] OR "Disease Management"[Mesh] OR self-management[tiab] OR disease management[tiab] OR patient education[tiab] OR patient engagement[tiab] OR medication adherence[tiab] OR healthcare delivery[tiab] OR clinical decision support[tiab] OR health coaching[tiab] OR symptom monitoring[tiab]))
Embase	('artificial intelligence'/exp OR 'machine learning'/exp OR 'natural language processing'/exp OR chatbot*:ti,ab,kw OR 'conversational agent*':ti,ab,kw OR 'virtual assistant*':ti,ab,kw OR 'digital assistant*':ti,ab,kw OR 'ai chatbot*':ti,ab,kw OR 'large language model*':ti,ab,kw OR llm*:ti,ab,kw OR chatgpt:ti,ab,kw OR bard:ti,ab,kw OR gemini:ti,ab,kw) AND ('chronic disease'/exp OR 'chronic illness':ti,ab,kw OR 'chronic disease*':ti,ab,kw OR diabetes:ti,ab,kw OR hypertension:ti,ab,kw OR 'cardiovascular disease*':ti,ab,kw OR 'heart failure':ti,ab,kw OR 'chronic kidney disease':ti,ab,kw OR ckd:ti,ab,kw OR copd:ti,ab,kw OR asthma:ti,ab,kw OR cancer:ti,ab,kw OR arthritis:ti,ab,kw OR obesity:ti,ab,kw) AND ('patient care'/exp OR 'disease management'/exp OR 'self care'/exp OR 'patient education'/exp OR self-management:ti,ab,kw OR disease management:ti,ab,kw OR patient engagement:ti,ab,kw OR medication adherence:ti,ab,kw OR symptom monitoring:ti,ab,kw OR health coaching:ti,ab,kw)
Scopus	TITLE-ABS-KEY(("artificial intelligence" OR AI OR chatbot* OR "AI chatbot*" OR "conversational agent*" OR "virtual assistant*" OR "digital assistant*" OR "intelligent virtual agent*" OR "large language model*" OR LLM* OR ChatGPT OR Bard OR Gemini)) AND TITLE-ABS-KEY(("chronic disease*" OR "chronic illness*" OR diabetes OR hypertension OR "cardiovascular disease*" OR "heart failure" OR "chronic kidney disease" OR CKD OR COPD OR asthma OR cancer OR arthritis OR obesity)) AND TITLE-ABS-KEY(("disease management" OR self-management OR "patient education" OR "patient engagement" OR "medication adherence" OR "clinical decision support" OR "health coaching" OR "symptom monitoring" OR healthcare))
Web of Science Core Collection	TS=(("artificial intelligence" OR AI OR chatbot* OR "AI chatbot*" OR "conversational agent*" OR "virtual assistant*" OR "digital assistant*" OR "intelligent virtual agent*" OR "large language model*" OR LLM* OR ChatGPT OR Bard OR Gemini)) AND TS=(("chronic disease*" OR "chronic illness*" OR diabetes OR hypertension OR "cardiovascular disease*" OR "heart failure" OR "chronic kidney disease" OR CKD OR COPD OR asthma OR cancer OR arthritis OR obesity)) AND TS=(("disease management" OR self-management OR "patient education" OR "patient engagement" OR "medication adherence" OR "clinical decision support" OR "health coaching" OR "symptom monitoring" OR healthcare))
